# Enhanced Stability and Charge Separation of InP by Assembling Al_2_O_3_ and Metallic Al for Photocatalytic Overall Water Splitting

**DOI:** 10.3390/molecules30183822

**Published:** 2025-09-20

**Authors:** Zhiquan Yin, Wenlong Zhen, Xiaofeng Ning, Zhengzhi Han, Gongxuan Lu

**Affiliations:** 1 State Key Laboratory of Low Carbon Catalysis and Carbon Dioxide Utilization, Lanzhou Institute of Chemical Physics, Chinese Academy of Sciences, Lanzhou 730000, China; 2 University of Chinese Academy of Sciences, Beijing 100049, China

**Keywords:** InP, Al, overall water splitting, visible light, stability

## Abstract

Photocatalytic hydrogen evolution from water is a sustainable approach to producing clean energy and promoting sustainable development. However, the low efficiency of photocatalysts under visible light and even near-infrared irradiation remains the current bottleneck in photocatalytic overall water splitting (POWS). Herein, we introduce an Al_2_O_3_/InP/Al photocatalyst with high efficiency and stability, which achieved an apparent quantum efficiency of 0.97% at 500 nm and operated for 10 h without significant performance decay. The high quantum efficiency and stability are attributed to the combination of the electron-rich Al reflective layer and Al_2_O_3_ protective layer coating, which synergistically facilitate charge separation. Additionally, the newly generated O_2_ from water over the Al_2_O_3_/InP/Al photocatalyst was removed under reduced pressure, thus inhibiting InP photocorrosion and enabling POWS under visible light irradiation. This study offers valuable insights for the development of efficient solar photocatalysts for water splitting.

## 1. Introduction

The overconsumption of fossil fuels necessitates a transition to renewable energy to mitigate climate change and achieve net-zero emissions [1]. Solar energy is a promising candidate due to its abundance and vast potential, but its intermittency requires efficient energy storage [2]. Hydrogen, an energy carrier with high mass-energy density and zero-carbon emissions upon combustion, is an ideal medium for this purpose [3]. Solar-driven POWS offers a sustainable pathway for producing green hydrogen, directly addressing the challenges of the energy crisis and environmental pollution [4,5,6]. Since Fujishima and Honda reported that photocatalytic water splitting occurs on TiO_2_ photoelectrodes [7], substantial efforts have been devoted to developing efficient photocatalysts for the hydrogen evolution reaction (HER). However, the solar-to-hydrogen (STH) efficiency remains low in most systems, far from the 10% threshold required for techno-economic viability [8]. A primary reason is the narrow spectral response of stable wide-bandgap materials, such as SrTiO_3_ [9], Ta_2_O_5_ [10], In_2_O_3_ [11], and Al_2_O_3_ [12], which are active in the UV region. Some visible-light-active semiconductors, such as SnS_2_ [13], CdS [14], ZnIn_2_S_4_ [15], and g-C_3_N_4_ [16], are often unstable in water and susceptible to photocorrosion. Notably, visible and infrared light counts for ~95% of AM1.5G, and harvesting these spectral regions may substantially improve the photocatalytic HER performance.

Indium phosphide (InP) is a narrow bandgap (~1.35 eV) semiconductor with efficient light absorption, high carrier mobility, and proven efficiency in thin-film solar cells (PCE up to 29.6%) [17,18,19,20]. These properties make it a promising candidate to address the spectral limitation. However, the low-valence state V-group element P is easily oxidized under light irradiation in aqueous solutions. Coating stable metal oxides, such as TiO_2_ [21], ZnO [22], and Al_2_O_3_ [23] onto III–V semiconductor surfaces, is a widely adopted strategy to prevent corrosion due to its low cost. Among them, Al_2_O_3_ is extensively used for interface passivation and photocatalytic HER, exhibiting excellent stability and charge transport [24,25,26,27]. Additionally, the efficiency of photoinduced carrier separation in photocatalysts is crucial for the performance of POWS. Recent advancements in metal–semiconductor junction architectures, with their unique charge separation and enhanced light-harvesting capabilities, offer a promising solution to this bottleneck [28,29]. In such systems, metallic layers (e.g., Al) act as reflective mirrors to prolong light pathways and improve photon utilization, facilitating efficient charge separation via Schottky junction formation [30,31].

In this work, we report the synthesis of efficient Al_2_O_3_/InP/Al photocatalysts for POWS via in situ growth of InP on the surface of electron-rich metallic Al, which is typically employed as a rear-surface reflector to enhance light absorption efficiency and simultaneously forms a metal-semiconductor junction that improves the separation efficiency of photogenerated carriers. To prevent the corrosion of InP, a thin Al_2_O_3_ shell coated on its surface significantly improves the stability of InP during photocatalytic water splitting. Additionally, a reduced-pressure system was employed to transfer hydrogen and oxygen generated within the reaction system, maintaining a low-pressure environment. This system significantly inhibits H_2_–O_2_ recombination on the surface of Al_2_O_3_/InP/Al, enhancing photocatalytic efficiency. This study demonstrated that the Al_2_O_3_/InP/Al photocatalyst exhibits an enhanced photocatalytic H_2_ production performance compared to bare InP, presents a high apparent quantum efficiency (AQE) (approximately 0.97% at 500 nm), and shows no significant performance decay after continuous illumination for 10 h. This work offers a potential avenue for solar energy conversion and storage by developing a novel approach to constructing an efficient and stable photocatalyst for water splitting.

## 2. Results and Discussion

### 2.1. XRD Analysis

**Figure 1:**
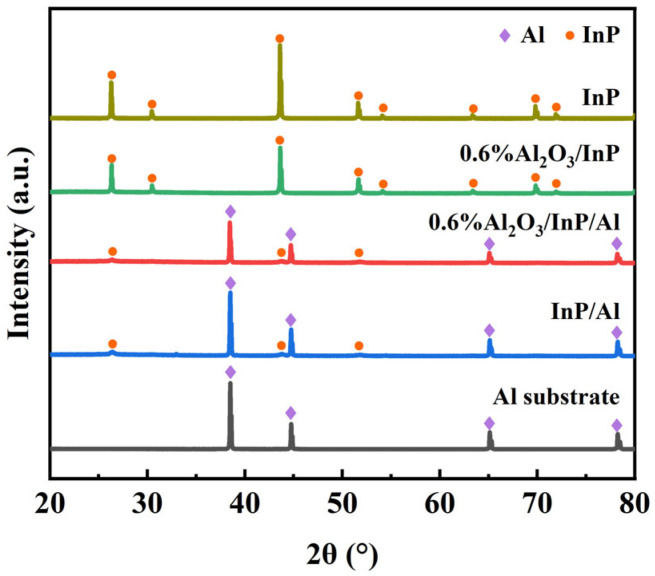
XRD patterns of Al substrate, InP, InP/Al, and 0.6%Al_2_O_3_/InP/Al. Cubic-phase Al and cubic-phase InP are marked by purple diamond and orange circle symbols, respectively.

The crystal structure of Al substrate, InP, InP/Al, and 0.6%Al_2_O_3_/InP/Al catalysts was analyzed using X-ray powder diffraction (XRD), and the results are shown in Figure 1. The Al substrate exhibits four intense diffraction peaks at 2θ = 38.5°, 44.7°, 65.1°, and 78.2°, correspond to the (111), (200), (220), and (311) crystal planes of the cubic phase Al according to JCPDS card No. 89-4037 (Figure S1) [32]. InP was used as the reference, which presents cubic phase InP, and the diffraction peaks at 26.3°, 30.4°, 43.6°, 51.6°, and 69.8° correspond to the (111), (200), (220), (311), and (331) crystal planes of cubic phase InP according to JCPDS card No. 73-1983 (Figure S2) [33]. After the growth of InP on the Al substrate, the InP/Al composite catalyst exhibits characteristic diffraction peaks of both InP and Al. Four strong diffraction peaks located at 38.5°, 44.7°, 65.1°, and 78.2° can be indexed to the (111), (200), (220), and (311) crystal planes of cubic Al. Three weak diffraction peaks are observed at 26.3°, 43.6°, and 51.6°, which can be assigned to the (111), (220), and (311) crystal planes of cubic InP. The above results confirm that an InP layer was successfully grown on the Al substrate via the phosphorization process. After constructing an Al_2_O_3_ protective layer on the InP/Al surface, the diffraction pattern of the 0.6%Al_2_O_3_/InP/Al composite was nearly identical to that of InP/Al, and no characteristic peaks of Al_2_O_3_ were found, which may be due to the lower surface Al_2_O_3_ content.

### 2.2. Morphological Analysis

**Figure 2:**
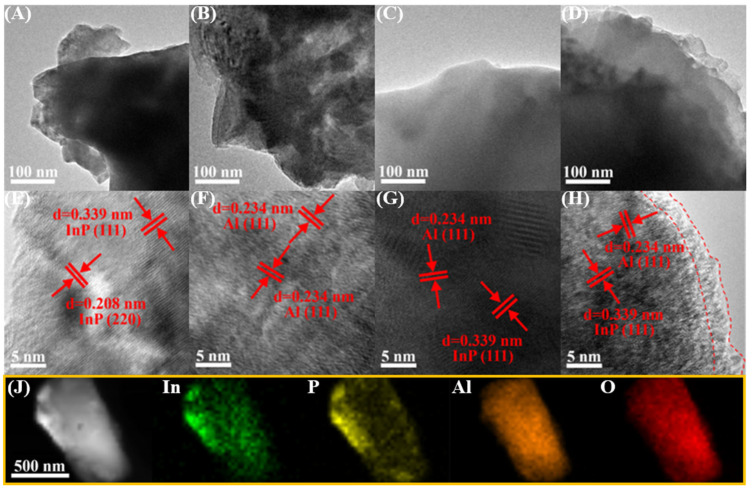
TEM images of (**A**) InP, (**B**) Al substrate, (**C**) InP/Al, and (**D**) 0.6%Al_2_O_3_/InP/Al; HR-TEM images of (**E**) InP, (**F**) Al substrate, (**G**) InP/Al, and (**H**) 0.6%Al_2_O_3_/InP/Al; (**J**) EDX elemental mapping images of 0.6%Al_2_O_3_/InP/Al. The double lines indicated by the arrows represent a set of crystal planes.

The morphology of Al substrate, InP, InP/Al, and 0.6%Al_2_O_3_/InP/Al was investigated using transmission electron microscopy (TEM) and high-resolution transmission electron microscopy (HR-TEM). Figure 2A shows the TEM image of InP surface morphology, which has smooth edges but irregularities. Figure 2E displays the HR-TEM image of InP, exhibiting two crystal planes of cubic InP, (111) and (220), with corresponding lattice spacings of 0.339 nm and 0.208 nm, respectively. The Al substrate exhibits irregular morphology with smooth edges (Figure 2B). The HR-TEM image corresponding to the Al substrate (Figure 2F) reveals the (111) plane of cubic Al (d_111_ = 0.234 nm). The InP/Al sample also exhibits an irregular morphology (Figure 2C). HR-TEM imaging (Figure 2G) presents two lattice fringes with lattice spacings of 0.339 nm and 0.234 nm. These spacings correspond to the (111) crystal plane of cubic InP and cubic Al, respectively. The presence of both sets of lattice fringes indicates that InP was grown on the Al surface. After loading Al_2_O_3_ on the surface of InP/Al, the surface of the 0.6%Al_2_O_3_/InP/Al sample became rougher compared to the InP/Al sample (Figure 2D), and the morphological change may be due to the growth of the Al_2_O_3_ protective layer on the InP/Al surface. In the HR-TEM image of the 0.6%Al_2_O_3_/InP/Al sample (Figure 2H), InP and Al can be observed, but no lattice fringes attributable to crystalline Al_2_O_3_ are detected. Notably, an amorphous shell is observed at the edge of 0.6%Al_2_O_3_/InP/Al, suggesting Al_2_O_3_ deposition [24]. Additionally, the Al, In, P, and O elements are uniform in 0.6%Al_2_O_3_/InP/Al, indicating that the 0.6%Al_2_O_3_/InP/Al photocatalyst has been successfully synthesized by coating the Al_2_O_3_ shell on the InP/Al surface (Figure 2J).

### 2.3. UV-Vis-NIR Analysis

**Figure 3:**
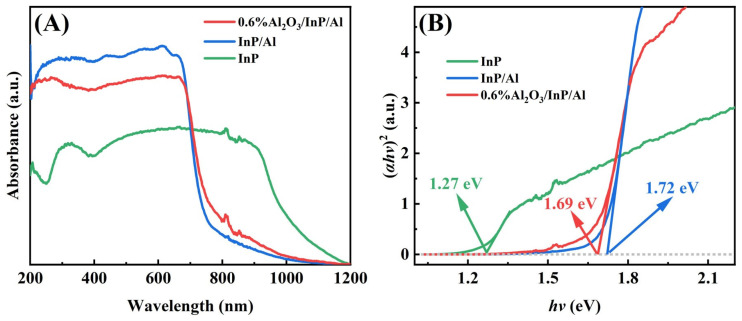
(**A**)The UV-Vis-NIR diffuse reflectance spectra and (**B**) bandgap of InP, InP/Al, and 0.6%Al_2_O_3_/InP/Al.

The optical properties of InP affected by Al and Al_2_O_3_ were analyzed by UV-vis-NIR diffuse reflectance spectra. Figure 3A shows the UV-vis-NIR diffuse reflectance spectra of InP, InP/Al, and 0.6%Al_2_O_3_/InP/Al. InP has a broad spectral response region and high absorption efficiency, making it an excellent light-harvesting material for solar energy conversion. For the InP/Al composite catalyst, the light absorption edge exhibits a blue shift, and absorption performance in 200–600 nm is significantly improved. The blue shift of InP/Al is caused by Al doping in InP during the synthesis of the In(OH)_x_/Al precursor. However, the enhanced absorption performance is attributed to Al reflecting photons that penetrate through InP, thereby improving light-harvesting efficiency. After loading Al_2_O_3_, the light absorption performance slightly decreases, which may be due to Al_2_O_3_ blocking the absorption of light, but the absorption edge of 0.6%Al_2_O_3_/InP/Al is close to InP/Al, indicating that Al_2_O_3_ coating has a weak impact on the light absorption region of InP/Al. The semiconductor bandgap can be derived by the following equation:

αhν = A (hν − E_g_)^n/2^ (1)

where α, ν, E_g_, and A represent the absorption coefficient, optical frequency, bandgap energy, and constant, respectively [34,35]. In addition, the exponent n depends on the electronic transition type of the semiconductor, whether direct transitions (n = 1) or indirect transitions (n = 4). The interband electronic transition mode of InP is a direct transition, and the bandgap energies of InP, InP/Al, and 0.6%Al_2_O_3_/InP/Al are 1.27, 1.72, and 1.69 eV, respectively (Figure 3B). These results are consistent with the absorption spectra, and the minor changes in bandgap values between InP/Al and 0.6%Al_2_O_3_/InP/Al indicate a weak impact on the light absorption range of InP/Al when Al_2_O_3_ loading.

### 2.4. XPS Analysis

**Figure 4:**
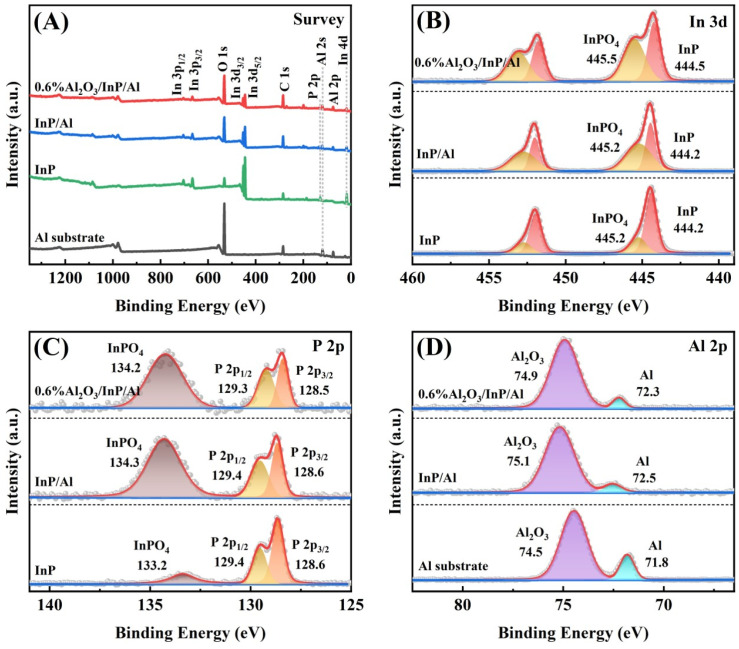
XPS spectra of InP, Al substrate, InP/Al, and 0.6%Al_2_O_3_/InP/Al: (**A**) Survey, (**B**) In 3d, (**C**) P 2p, and (**D**) Al 2p.

The surface chemical states of the prepared samples were investigated using X-ray photoelectron spectroscopy (XPS), with all spectra calibrated against the C 1s peak (Figure S3). The survey spectra of Al substrate, InP, InP/Al, and 0.6%Al_2_O_3_/InP/Al confirm the existence of Al, In, P, and O in the 0.6%Al_2_O_3_/InP/Al catalyst, in agreement with EDS results (Figure 4A). Figure 4B shows the In 3d spectra of InP, InP/Al, and 0.6%Al_2_O_3_/InP/Al. The binding energy ranges of 442.5–447.5 eV and 450.0–455.0 eV are assigned to the In 3d_5/2_ and In 3d_3/2_ peaks, respectively. The In 3d spectra for all samples can be deconvoluted into two distinct components, corresponding to InP and InPO_4_. In the InP sample, the In 3d peaks at 444.2 eV (In 3d_5/2_) and 452.0 eV (In 3d_3/2_) are assigned to InP, while those at 445.2 eV (In 3d_5/2_) and 452.7 eV (In 3d_3/2_) are assigned to InPO_4_ [36,37]. The binding energies of InP and InPO_4_ species in the InP/Al sample are consistent with those in pure InP, but the InPO_4_ content in InP/Al increased. This increase is attributed to the partial oxidation of InP during phosphidation. With the Al_2_O_3_ loading onto InP/Al, the content of InPO_4_ on 0.6%Al_2_O_3_/InP/Al surface further increased, and the binding energies of both InP and InPO_4_ shifted towards higher binding energies by 0.3 eV, indicating successful loading of Al_2_O_3_ on the surface of InP/Al. Figure 4C presents the P 2p spectra of InP, InP/Al, and 0.6%Al_2_O_3_/InP/Al. In the bare InP, the characteristic peaks of P 2p_3/2_ and P 2p_1/2_ corresponding to P^3−^ in InP emerged at 128.6 and 129.4 eV, respectively, while the peak at 133.2 eV belongs to P^5+^ in InPO_4_ [24,38,39,40]. The peak position of P^3−^ in InP/Al did not change compared with InP. In contrast, the P^5+^ peak shifted towards high binding energy by ~1.1 eV, and the relative content significantly increased, indicating oxidation during phosphidation. After Al_2_O_3_ loading, the peaks of P^3−^ and P^5+^ in 0.6%Al_2_O_3_/InP/Al shifted to higher binding energies by 0.1 eV, demonstrating electron transfer from the InP/Al to Al_2_O_3_. Figure 4D exhibits the Al 2p spectra of Al substrate, InP/Al, and 0.6%Al_2_O_3_/InP/Al. The Al 2p spectrum of the Al substrate exhibits two peaks at the binding energies of 71.8 eV and 74.5 eV, corresponding to the metallic state Al and surface Al_2_O_3_, respectively [30,31,41,42]. The metallic state Al and surface Al_2_O_3_ of InP/Al shift upward by 0.6 ± 0.1 eV compared to Al substrate, suggesting InP is deposited on metallic Al surfaces and charge transfer from Al to InP [41,43,44,45]. With Al_2_O_3_ coating, the binding energies of metallic Al and Al_2_O_3_ decreased by 0.2 eV, indicating that the Al_2_O_3_ passivation layer was successfully loaded onto the InP/Al surface. Figure S4 displays the O 1s spectra of InP, Al substrate, InP/Al, and 0.6%Al_2_O_3_/InP/Al. The binding energy at 531.7 eV in InP is attributed to the PO_4_^3−^ in InPO_4_, while the binding energy at 533.4 eV corresponds to the adsorbed O on the surface of InP [46,47,48]. In the Al sample, the O 1s characteristic peak is observed at 531.9 eV. The O 1s spectrum of InP/Al can be deconvoluted into three peaks of surface adsorbed O at 533.4 eV, lattice O in Al_2_O_3_ at 532.1 eV, and PO_4_^3−^ at 531.9 eV. After loading Al_2_O_3_ onto InP/Al, the O 1s peaks of Al_2_O_3_ and PO_4_^3−^ within 0.6%Al_2_O_3_/InP/Al shifted to lower binding energies by 0.1 eV, and the intensity of lattice O in Al_2_O_3_ increased compared to InP/Al, which was attributed to the Al_2_O_3_ loading.

### 2.5. Photocatalytic Hydrogen Production Performance

The photocatalytic HER performance of InP/Al was investigated under visible light irradiation in a reduced-pressure system. Figure S5 shows the HER activity of InP/Al photocatalysts at varied Cl (in InCl_3_) to NH_3_ (Cl:NH_3_) molar ratios. As the NH_3_ content increases, the activity initially rises, reaching a maximum at a Cl:NH_3_ molar ratio of 3:1, followed by a gradual decline. This trend is attributed to the reaction between protons (produced via In^3+^ hydrolysis in aqueous solution) and the Al substrate, which generates an interfacial buffer layer. This buffer layer reduces lattice mismatch between the Al substrate and the InP film, enhances carrier separation and transport, and thus improves photocatalytic efficiency [49]. Figure S6 displays the influence of NHP mass on the HER performance of InP/Al during phosphatizing. HER activity initially increases with NHP mass, peaking at an NHP:Al mass ratio of 4:1, which is due to optimized InP crystallite formation. Beyond this mass ratio, activity declines progressively. This decrease may arise from PH_3_ gas (generated by excess NHP) decomposing on InP surfaces to form passivating phosphorus-rich species, which inhibit charge transfer. Figure S7 illustrates the influence of the initial Al:In mass ratio on the photocatalytic HER performance of InP/Al. The HER rate initially increases with InP mass content but declines beyond a threshold, peaking at an Al:In mass ratio of 4:1. When the Al:In mass ratio is below 4:1, InP partially exposes the Al substrate. Conversely, excessive InP loading (ratios > 4:1) leads to insufficient light absorption and impaired photoexcited charge conductivity near the Al interface. Furthermore, the actual mass ratio of Al to In in InP/Al at the optimal HER activity was confirmed to be 8.1:1 (Figure S8). This discrepancy between the initial mass ratio and the final atomic composition is attributed to losses of indium during the phosphidation processes.

**Figure 5:**
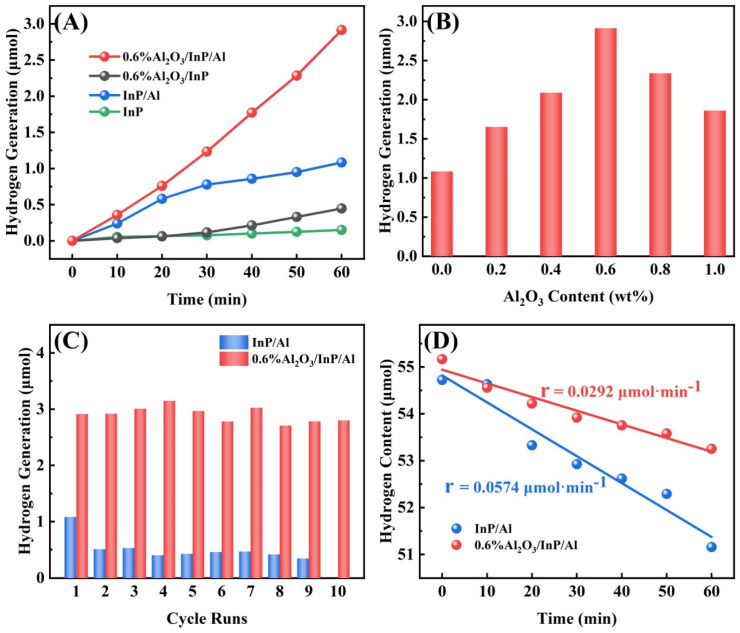
(**A**) The hydrogen production performance of InP, InP/Al, 0.6%Al_2_O_3_/InP and 0.6%Al_2_O_3_/InP/Al, (**B**) the photocatalytic hydrogen production of Al_2_O_3_/InP/Al at different Al_2_O_3_ loading content (calculated based on Al content), (**C**) the hydrogen production stability of InP/Al and 0.6%Al_2_O_3_/InP/Al, and (**D**) the H_2_–O_2_ recombination reaction of InP/Al and 0.6%Al_2_O_3_/InP/Al.

Comparative experiments were conducted to confirm the function of an Al substrate and an Al_2_O_3_ protection layer in the 0.6%Al_2_O_3_/InP/Al photocatalyst. As displayed in Figure 5A, Bare InP shows negligible hydrogen production, due to significant electron-hole recombination loss and critical corrosion of the InP. After supporting InP on the Al substrate, the HER activity of InP/Al composite significantly improved, 7.3-fold higher than that of bare InP. But severe corrosion of InP/Al remains. Similarly, the HER activity of InP increased threefold after loading with Al_2_O_3_, indicating that corrosion of InP can be suppressed by coating with Al_2_O_3_. By integrating these advantages, the 0.6%Al_2_O_3_/InP/Al photocatalyst presents the highest HER performance of 145.64 μmol·g^−1^·h^−1^, a 19.57-fold enhancement compared with the pristine InP catalyst. These results indicate that charge separation and corrosion resistance were significantly improved by combining InP with the Al substrate and a coated Al_2_O_3_ protection layer. We further optimized the Al_2_O_3_ loading content, and the results are shown in Figure 5B. As the Al_2_O_3_ loading increased, the photocatalytic activity of Al_2_O_3_/InP/Al improved, reaching an optimum at 0.6 wt% loading. Beyond this threshold, further increases in Al_2_O_3_ loading led to a decline in HER activity. This decline may stem from attenuation of light absorption by InP due to the thicker Al_2_O_3_ layer (Figure S9), thereby reducing the concentration of photoexcited carriers within the catalyst. Figure 5C compares the stability of InP/Al and 0.6%Al_2_O_3_/InP/Al catalysts in a long-term hydrogen production test. Notably, the HER activity of InP/Al declines sharply with cycling, indicating severe photocorrosion. By comparison, the 0.6%Al_2_O_3_/InP/Al sample operated for 10 h without significant decay, demonstrating higher efficiency and superior stability. The enhanced performance originates from the Al_2_O_3_ protective layer, which simultaneously accelerates the transfer of photoinduced electrons and inhibits InP degradation by isolating the catalyst from direct water contact, thereby significantly improving the stability and HER performance. The morphology and structure analysis after reaction also confirm the stability of the 0.6%Al_2_O_3_/InP/Al photocatalyst (Figures S10 and S11). In addition, the HER performance of the 0.6%Al_2_O_3_/InP/Al photocatalyst was compared under reduced-pressure and atmospheric pressure systems (Figure S12). The 0.6%Al_2_O_3_/InP/Al photocatalyst exhibits higher HER activity under reduced pressure than under atmospheric pressure conditions. This enhancement arises because reduced pressure removes newly generated O_2_ during the reaction, prevents oxygen-induced photocorrosion, and suppresses H_2_–O_2_ recombination on the catalyst surface (Figure 5D).

### 2.6. Isotope Tracing Experiment and AQE Analysis

**Figure 6:**
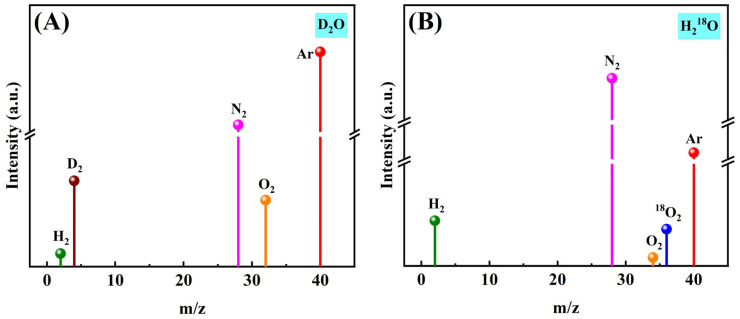
MS spectra of visible light photocatalytic decomposition of (**A**) D_2_O and (**B**) H_2_^18^O over 0.6%Al_2_O_3_/InP/Al catalyst.

To identify the productions obtained from photocatalytic water splitting, isotope tracing experiments were performed over 0.6%Al_2_O_3_/InP/Al photocatalyst under visible light irradiation. The observed signal at mass-to-charge ratio (*m*/*z*) of 4 is attributed to D_2_ generated by the photocatalytic splitting of D_2_O (Figure 6A). Similarly, the signal at *m*/*z* = 36 in Figure 6B confirms that ^18^O_2_ originates from photocatalytic decomposition of H_2_^18^O over the 0.6%Al_2_O_3_/InP/Al catalyst, demonstrating its oxygen evolution activity. These results collectively verify that visible-light-driven overall water splitting produces H_2_ and O_2_ on a 0.6%Al_2_O_3_/InP/Al photocatalyst. Figure S13 presents the AQE for overall water splitting over the 0.6%Al_2_O_3_/InP/Al catalyst across wavelengths from 500 to 750 nm. The AQE decreases with increasing wavelength, exhibiting strong wavelength dependence. A maximum AQE of 0.97% is observed at 500 nm, while the AQE at 750 nm remains 0.45%, suggesting significant photocatalytic activity in the visible light region. Additionally, Table S1 lists the AQE at 500 nm for the POWS reaction is comparable to those reported in recent studies for similar heterostructures, highlighting the competitiveness of our catalyst system.

### 2.7. Electrochemical Analysis

**Figure 7:**
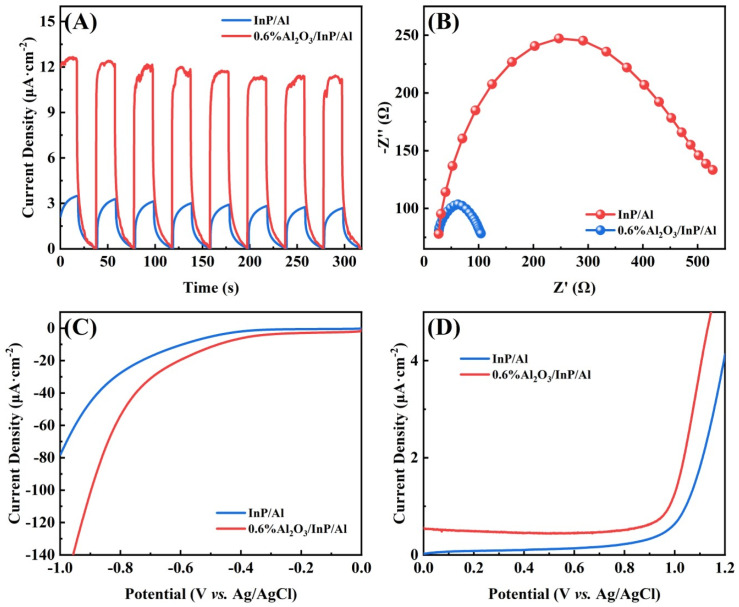
(**A**) Transient photocurrent responses, (**B**) electrochemical impedance spectroscopy Nyquist plots, (**C**) cathodic polarization curve, and (**D**) anodic polarization curve of InP/Al and 0.6%Al_2_O_3_/InP/Al.

The photocatalytic HER performance and photoinduced carrier separation efficiency were evaluated using an electrochemical method. Figure 7A exhibits the transient photocurrent response of InP/Al and 0.6%Al_2_O_3_/InP/Al under visible light pulse irradiation. InP/Al exhibits a lower photocurrent density and decreases significantly with extended irradiation time, implying photocorrosion of the catalyst. In contrast, 0.6%Al_2_O_3_/InP/Al exhibits a higher photocurrent density, about four times higher than InP/Al, indicating that the Al_2_O_3_ passivation layer enhances charge separation, prolongs carrier lifetime, and significantly improves stability [27,50]. Subsequently, electrochemical impedance spectroscopy (EIS) was applied to analyze the charge transfer properties of photoexcited carriers in photocatalysts. Figure 7B displays the EIS spectra of the as-prepared catalysts related to charge transfer at high-frequency regions. The large EIS semicircle diameter of InP/Al indicates high charge transfer resistance. The resistance of carrier transfer in 0.6%Al_2_O_3_/InP/Al is significantly reduced compared to InP/Al, indicating that the charge transfer rate can be remarkably enhanced by coating Al_2_O_3_ layers [51,52]. The hydrogen and oxygen evolution performance of InP/Al and 0.6%Al_2_O_3_/InP/Al was analyzed using linear sweep voltammetry. Figure 7C presents the cathodic polarization curves of InP/Al and 0.6%Al_2_O_3_/InP/Al catalysts, and the cathodic current densities observed in the range of −0.2 to −1.0 V (vs. Ag/AgCl) correspond to the generation of H_2_. 0.6%Al_2_O_3_/InP/Al catalyst presents an enhanced HER current density and a lower overpotential than InP/Al [53,54]. The anodic polarization curve of the catalysts is shown in Figure 7D, and the observed anodic current densities in the range of 1.0–1.2 V (vs. Ag/AgCl) correspond to the generation of O_2_. Compared with InP/Al, the oxygen evolution overpotential of 0.6%Al_2_O_3_/InP/Al catalyst is reduced, and the oxygen evolution current density is improved [55,56]. The above results indicate that 0.6%Al_2_O_3_/InP/Al is an excellent photocatalyst for overall water splitting. Furthermore, we investigated the charge carrier separation and transport properties of 0.6%Al_2_O_3_/InP/Al prepared at Cl (in InCl_3_) to NH_3_ molar ratios of 1:1 and 3:1, and the results are shown in Figures S14 and S15. The higher photocurrent density and smaller EIS semicircle diameter observed for the 0.6%Al_2_O_3_/InP/Al prepared at Cl:NH_3_ = 3:1 indicate significantly enhanced carrier separation and transfer efficiency. This improvement is attributed to a buffer layer formed between the Al substrate and InP, which reduces their lattice mismatch, thereby contributing to the photocatalytic water-splitting performance.

### 2.8. Photoluminescence Spectra Analysis

**Figure 8:**
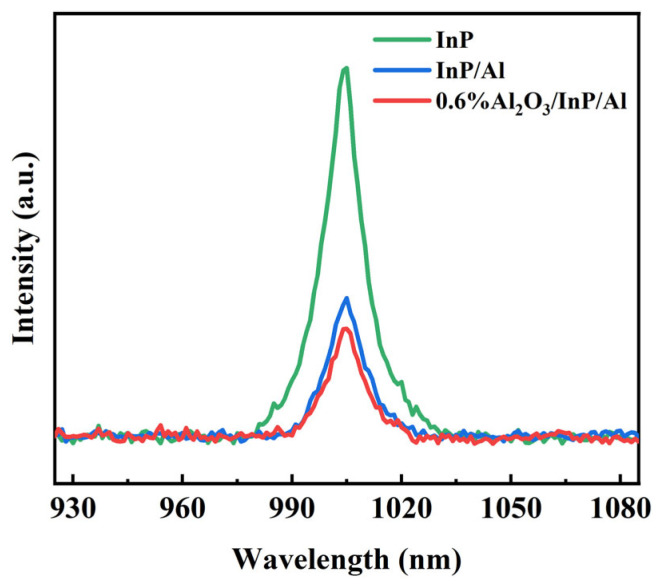
PL spectra of InP, InP/Al, and 0.6%Al_2_O_3_/InP/Al catalysts excited at 600 nm.

To further analyze the carrier separation and recombination efficiency of the catalysts, photoluminescence (PL) spectroscopy was performed. Figure 8 shows the PL emission spectra of InP, InP/Al, and 0.6%Al_2_O_3_/InP/Al with an excitation wavelength of 600 nm at room temperature. InP displayed the highest intensity of PL signal, indicating rapid recombination of photoinduced carriers. The PL intensity of InP, InP/Al, and 0.6%Al_2_O_3_/InP/Al decreased gradually, while the 0.6%Al_2_O_3_/InP/Al catalyst exhibited the lowest fluorescence intensity. The fluorescence intensity decreased significantly after the combination of InP and Al, which is attributed to the formation of a metal-semiconductor junction between InP and Al that effectively facilitates charge carrier separation [31,44]. Upon loading Al_2_O_3_ onto the InP/Al surface, the fluorescence intensity further decreased, due to the thin Al_2_O_3_ layer passivating the InP/Al interface, effectively reducing surface defects and thus suppressing surface recombination [57,58]. The corresponding average fluorescence lifetimes of InP, InP/Al, and 0.6%Al_2_O_3_/InP/Al are listed in Table 1. The fluorescence lifetimes of InP, InP/Al, and 0.6%Al_2_O_3_/InP/Al increased successively, indicating that the migration of carriers as well as electron-hole separation are enhanced by the Al substrate and Al_2_O_3_ thin layers. These results demonstrate that the composite of Al and Al_2_O_3_ can promote the separation and transfer of photoinduced carriers [51,59], whereas Al primarily enhances carrier separation, and Al_2_O_3_ suppresses the corrosion of InP, thereby achieving efficient photocatalytic water-splitting performance.

### 2.9. Mechanism Analysis

**Scheme 1:**
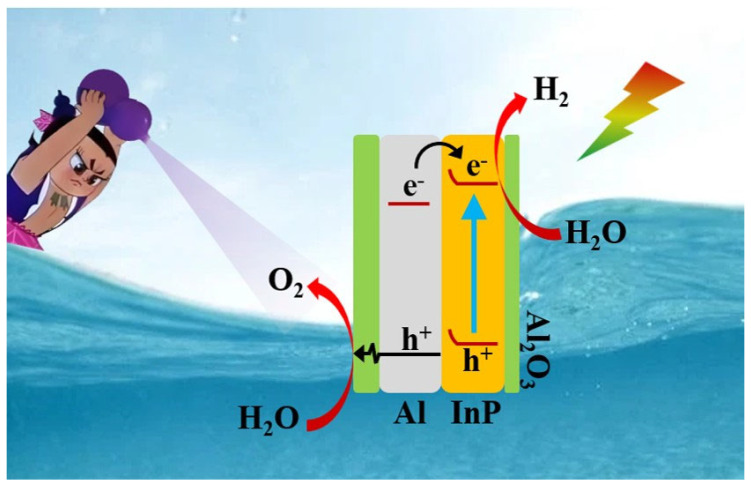
Mechanism of POWS over Al_2_O_3_/InP/Al using a reduced-pressure system under visible illumination.

The experimental results and characterization data form the basis for the proposed PWOS mechanism on the Al_2_O_3_/InP/Al catalyst, which is depicted in Scheme 1. Under visible light irradiation, electrons within the Al substrate are transferred to the conduction band of InP and participate in the reaction of water reduction to generate H_2_ [60]. The holes generated in the valence band of InP are transferred to the Al surface, followed by tunneling through a thin layer of Al_2_O_3_ or transferring to the surface through the abundant Al^3+^ cation vacancy network in the Al_2_O_3_ for water oxidation to generate O_2_ [27,61]. The Schottky barrier at the interface between InP and Al promotes the separation of InP photoexcited carriers and prolongs the photoexcited carrier lifetime. Simultaneously, the thin Al_2_O_3_ layer protects InP/Al from photocorrosion and reduces defect-induced surface recombination. Finally, the dissolved oxygen in water was expelled from the reactor using a reduced-pressure system. Consequently, Al_2_O_3_/InP/Al can realize POWS with excellent performance.

## 3. Materials and Methods

### 3.1. Materials

This study used aluminum powder (Al, >99%, Shanghai Aladdin Biochemical Technology Co., Ltd., Shanghai, China) as a substrate. Indium chloride (InCl_3_, >99.99%, Shanghai Aladdin Biochemical Technology Co., Ltd., Shanghai, China), aluminum chloride (AlCl_3_, >99%, Shanghai Aladdin Biochemical Technology Co., Ltd., Shanghai, China), sodium hypophosphite (NaH_2_PO_2_, abbreviated as NHP, >99.5%, Shanghai Macklin Biochemical Co., Ltd., Shanghai, China), and ammonia solution (NH_3_·H_2_O, 28–30 wt%, Shanghai Macklin Biochemical Co., Ltd., Shanghai, China) were used as the indium, aluminum, phosphorus, and alkali sources, respectively. Commercial indium phosphide (InP, 99.998%, Shanghai Macklin Biochemical Co., Ltd., Shanghai, China) was used as a reference material for comparison. All reagents were used without further purification.

### 3.2. Preparation of Photocatalysts

**Scheme 2:**
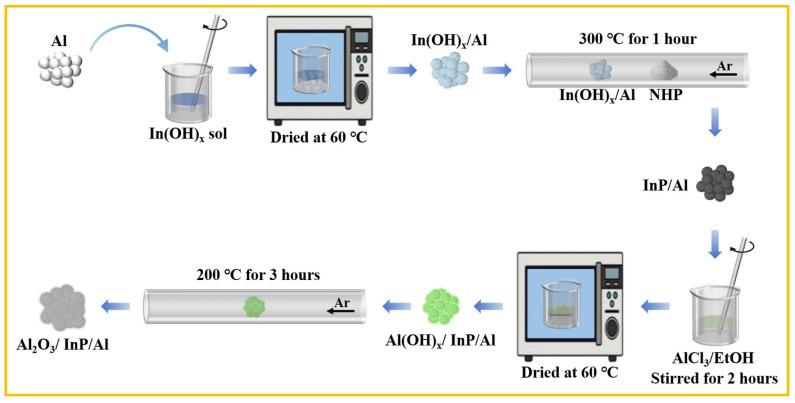
Process diagram for preparing Al_2_O_3_/InP/Al photocatalysts.

The synthesis of the Al_2_O_3_/InP/Al photocatalyst is shown in Scheme 2, while detailed experimental procedures are described below.

#### 3.2.1. Preparation of InP/Al Sample

Firstly, the In(OH)_x_ sol was prepared by adjusting the molar ratios between Cl (in InCl_3_) and NH_3_. The In(OH)_x_ sol and Al substrate were mixed at specific Al:In mass ratios, and the mixture was dried at 60 °C to produce In(OH)_x_/Al precursor. NHP and the In(OH)_x_/Al precursor (at designated mass ratios) were positioned upstream and downstream of the inlet in a quartz tube furnace, respectively. After rinsing the tube furnace with argon gas flow (50 mL·min^−1^) to exclude air, the temperature was raised to 300 °C at a rate of 2 °C·min^−1^ and maintained at a constant temperature for 1 h to obtain InP/Al. The sample was collected after being cooled to room temperature for future utilization.

#### 3.2.2. Preparation of Al_2_O_3_/InP/Al Sample

To prepare the Al_2_O_3_/InP/Al photocatalyst, AlCl_3_ corresponding to 0.2 wt% Al_2_O_3_ (calculated based on Al content) was dissolved in 10 mL of anhydrous ethanol, and then 50 mg of InP/Al was added. The solution was continuously stirred at room temperature for 2 h and then dried at 60 °C. Subsequently, the dried sample was heated to 200 °C at a rate of 5 °C·min^−1^ in an argon gas flow and kept at a constant temperature for 3 h. Al_2_O_3_/InP/Al photocatalysts with Al_2_O_3_ loadings of 0.4 wt%, 0.6 wt%, 0.8 wt%, and 1.0 wt% were prepared using the same method.

### 3.3. Photocatalytic H_2_ Evolution Experiments

All photocatalytic hydrogen production experiments were carried out under a reduced-pressure system. The detailed procedure is as follows: 20 mg of the catalyst was evenly dispersed on the bottom of a 140 mL sealed reactor containing 50 mL deionized water, which was equipped with a flat quartz window. The system was purged with high-purity argon (15 mL·min^−1^), and the evolved gases were collected by a collection tube with a silicone seal. The total volume of mixed gas was quantified via water displacement. The Xenon lamp light source (incident light power: 200 mW·cm^−1^, Beijing NBET Technology Co., Ltd., Beijing, China) was equipped with a 420 nm cut-off filter. Gas chromatography (Agilent 6820, TCD, 13X Column, Ar carrier gas, Agilent Technologies (Shanghai) Co., Ltd., Shanghai, China) was employed for qualitative and quantitative analysis of the gas mixture in the collection tube during the water-splitting process. The stability experiment for photocatalytic hydrogen evolution was carried out as follows. After the first photocatalytic reaction under visible light irradiation, the collection tube was filled with water again without further treatment of the photocatalyst. Subsequently, the mixed gas was recollected to fill the tube, and this procedure was repeated for each cycle.

Under the same photocatalytic reaction conditions, monochromatic light was obtained using a bandpass filter (center wavelengths: 500, 550, 600, 650, 700, and 750 nm) for the AQE measurement of the catalyst. The number of incident photons was determined using a radiometer with a silicon photodetector (FU 100). The reactor was irradiated with monochromatic light for 1 h, and the hydrogen yield was quantified via gas chromatography. The AQE was calculated using the following equation:

(2) $$AQE=2\times \frac{the\;number\;of\;hydrogen\;molecules}{the\;number\;of\;incident\;photons}\times 100\%$$

## 4. Conclusions

The synthesized Al_2_O_3_/InP/Al composite photocatalyst demonstrated excellent photocatalytic activity and remarkable stability for PWOS. The Al_2_O_3_ protective layer on the surface of InP could rapidly transfer photogenerated holes and protect InP from photocorrosion. As a light reflection layer, the electron-rich Al can effectively separate photoinduced carriers and enhance the light absorption performance of InP. Furthermore, to achieve the POWS and long-term stability, the reaction system was maintained under reduced pressure to remove dissolved oxygen. This effectively suppressed the H_2_–O_2_ recombination reverse reaction and minimized photocorrosion of InP. Thus, the Al_2_O_3_/InP/Al photocatalyst can achieve efficient and stable POWS under visible light illumination. This work provides a viable strategy for designing and manufacturing efficient and durable InP-based photocatalysts for solar-driven green hydrogen production.

## Figures and Tables

**Table 1:** Fluorescence lifetimes of InP, InP/Al, and 0.6%Al_2_O_3_/InP/Al composite.

Samples	Lifetime (ns)	Pre-Exponential Factors B	Average Lifetime (ns)	Residual Weighting (X^2^)
InP	T_1_	6.60	2.34	0.9951
	T_2_	0.95		
InP/Al	T_1_	13.91	4.14	0.9987
	T_2_	1.01		
0.6%Al_2_O_3_/InP/Al	T_1_	17.42	5.23	1.0404
	T_2_	1.04		

## Data Availability

Data are contained within the article and Supplementary Materials.

## Supplementary Materials

The following supporting information can be downloaded at https://www.mdpi.com/article/10.3390/molecules30183822/s1, Figure S1: XRD patterns of Al substrate and corresponding JCPDS card; Figure S2: XRD patterns of InP and corresponding JCPDS card; Figure S3: XPS C 1s spectra of InP, Al substrate, InP/Al, and 0.6%Al_2_O_3_/InP/Al; Figure S4: XPS O 1s spectra of InP, Al substrate, InP/Al, and 0.6%Al_2_O_3_/InP/Al; Figure S5: The photocatalytic hydrogen production of InP/Al prepared by adjusting the molar ratios between Cl (in InCl_3_) and NH_3_; Figure S6: The photocatalytic hydrogen production of InP/Al is affected by NHP:Al mass ratio; Figure S7: The photocatalytic hydrogen production of InP/Al is affected by Al:In mass ratio; Figure S8: The content of Al and In in InP/Al; Figure S9: (A) TEM and (B) HRTEM images of 1.0%Al_2_O_3_/InP/Al photocatalyst; Figure S10: (A) TEM and (B) HRTEM images of 0.6%Al_2_O_3_/InP/Al photocatalyst after 10 h of photocatalytic reaction; Figure S11: XRD patterns of 0.6%Al_2_O_3_/InP/Al photocatalyst after 10 h of photocatalytic reaction; Figure S12: The photocatalytic hydrogen production activity under reduced pressure over 0.6%Al_2_O_3_/InP/Al photocatalyst; Figure S13: The AQE of 0.6%Al_2_O_3_/InP/Al catalyst at different wavelengths; Figure S14: The transient photocurrent response of 0.6%Al_2_O_3_/InP/Al with varying Cl (in InCl_3_) to NH_3_ molar ratios (1:1 and 3:1); Figure S15: The EIS spectra of 0.6%Al_2_O_3_/InP/Al with varying Cl (in InCl_3_) to NH_3_ molar ratios (1:1 and 3:1). Table S1: The AQEs of hydrogen production using In-based and Al-based photocatalysts. References [27,62,63,64,65,66,67] are cited in the supplementary materials.
