# Supplementary material for: Enhanced Stability and Charge Separation of InP by Assembling Al2O3 and Metallic Al for Photocatalytic Overall Water Splitting
Source: Molecules. 2025 Sep 20;30(18):3822. doi: 10.3390/molecules30183822 (PMC12472304; doi:10.3390/molecules30183822)
Supplement: Supplementary file 1 [file molecules-30-03822-s001.zip › Supporting Information.pdf]

Supporting information

Enhanced stability and charge separation of InP by assembling Al_2O_3 and metallic Al for photocatalytic overall water splitting

Zhiquan Yin[1,2], Wenlong Zhen[1*], Xiaofeng Ning[1], Zhengzhi Han[1], Gongxuan Lu[1*]

[1] State Key Laboratory of Low Carbon Catalysis and Carbon Dioxide Utilization, Lanzhou Institute of Chemical Physics, Chinese Academy of Sciences, Lanzhou 730000, China
[2] University of Chinese Academy of Sciences, Beijing 100049, China
* Correspondence: gxlu@lzb.ac.cn

1. Experimental sections

1.1 Characterization of the catalysts

The crystallographic information of the catalyst was recorded using a Rigaku B/Max-RB X-ray powder diffractometer (XRD) equipped with a Ni-filtered Cu Kα source, scanning at a $0.01°\cdot s^{-1}$ rate over a 2θ range of 10-80°. X-ray photoelectron spectroscopy (XPS) analysis was performed using a VG Scientific ESCALAB210-XPS spectrometer with an Al Kα X-ray source. The Tecnai-G2-F30 field-emission transmission electron microscope (TEM) was used to obtain TEM and high-resolution TEM (HR-TEM) images of the sample at an acceleration voltage of 300 kV. The photoluminescence (PL) spectra and time-resolved photoluminescence (RT-PL) spectra of the samples were obtained using a Hitachi F-4500 photoluminescence spectrometer. The UV-Vis-NIR diffuse reflectance spectrum (DRS) of the sample was collected by the PerkinElmer Lambda 950 UV/vis/NIR spectrometer with $BaSO_4$ powder as the internal standard.

1.2 Recombination experiments of H_2 and O_2

A sealed reactor with a silicone rubber septum at the top was used for detecting H_2-O_2 recombination experiments. The experimental details are as follows: In the reactor, 20 mg of photocatalyst was ultrasonically dispersed in 100 mL of deionized water. High-purity Ar was purged through the reactor to ensure air exclusion. After that, H_2 (1.2 mL) and O_2 (0.6 mL) were injected into the reactor, simulating the H_2-O_2 recombination reaction under dark conditions. The recombination rate was calculated based on the average hydrogen consumption during the reaction.

1.3 Photocatalytic splitting D_2O and $H_2{}^{18}O$ isotope-labeled experiments

Isotope tracer experiments were conducted in a mini-reactor. Typically, 10 mg of the catalyst was dispersed within a sealed quartz reactor containing 10 mL of D_2O or 2 mL of $H_2{}^{18}O$. After ultrasonic treatment for 30 minutes, the system was purged with argon gas for 30 minutes to exclude air from the reactor. After visible light irradiation for 3 hours, the gaseous products were analyzed by MS (TILON LC-D200M mass spectrometer, equipped with a high-vacuum sampling valve and quartz glass capillary tube, with capillary temperature maintained at 120 °C).

1.4 Photoelectrochemical performance

Photoelectrochemical data were collected on an electrochemical analyzer (CHI660E). Photoelectrochemical experiments were performed in a standard three-electrode electrolytic cell equipped with a 1.2 cm diameter quartz window facing the working electrode. A platinum plate served as the counter electrode, and a silver/silver chloride (Ag/AgCl) electrode was used as the reference electrode. The working electrode was prepared by depositing the sample suspension onto a 1 × 1 cm^2 area of cleaned indium tin oxide (ITO) glass, details were as follows: 10 mg of Al_2O_3/InP/Al was dispersed in 5 mL deionized water, sonicated for 30 minutes, then 100 μL of the suspension was drop-cast onto a cleaned ITO glass substrate and dried at room temperature to form a film. The photocurrent-time curve (I-t curve) was recorded under open-circuit voltage conditions. The linear sweep voltammetry curve (LSV) was measured with a scan rate of 0.5 $mV\cdot s^{-1}$. A 300 W xenon lamp with a 420 nm cut-off filter served as the excitation source, and the electrolyte was 0.1 M Na_2SO_4 solution.

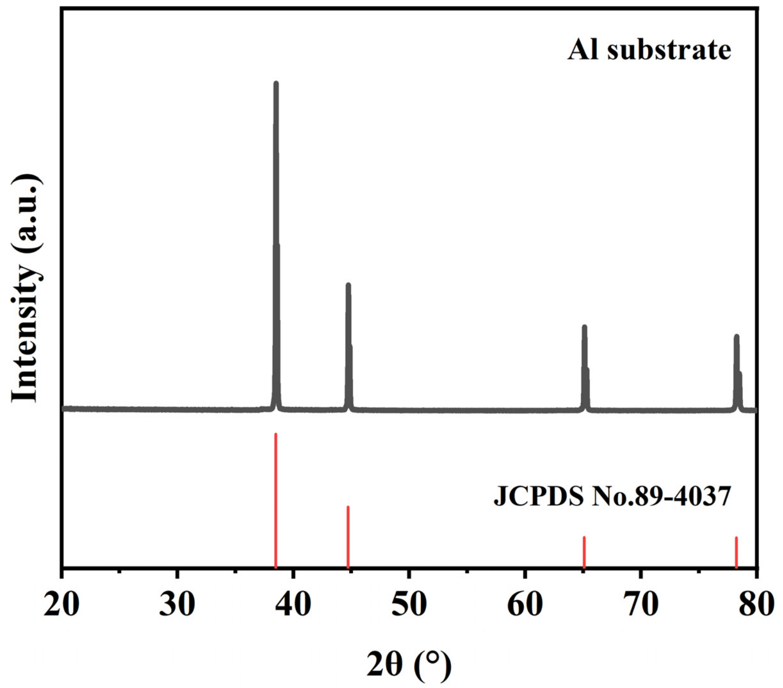

Figure S1. XRD patterns of Al substrate and corresponding JCPDS card.

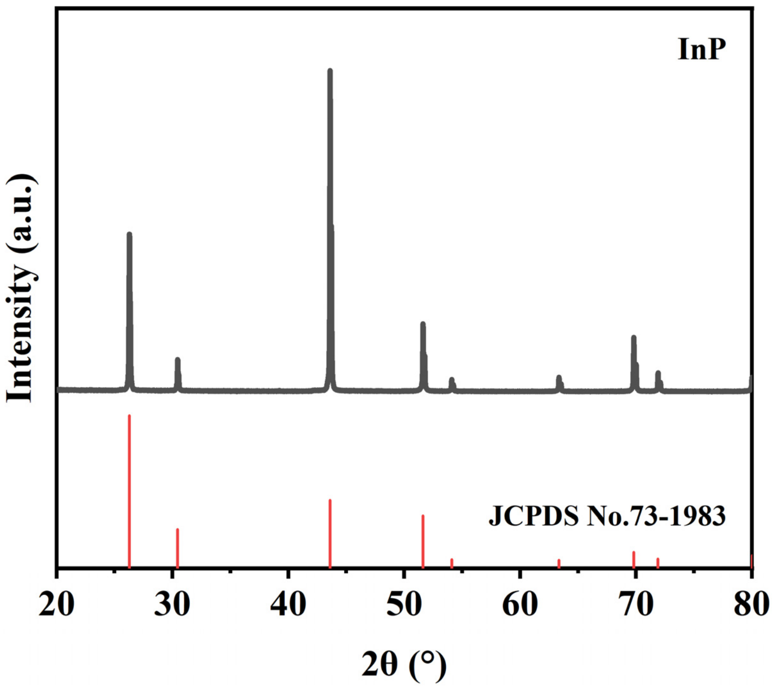

Figure S2. XRD patterns of InP and corresponding JCPDS card.

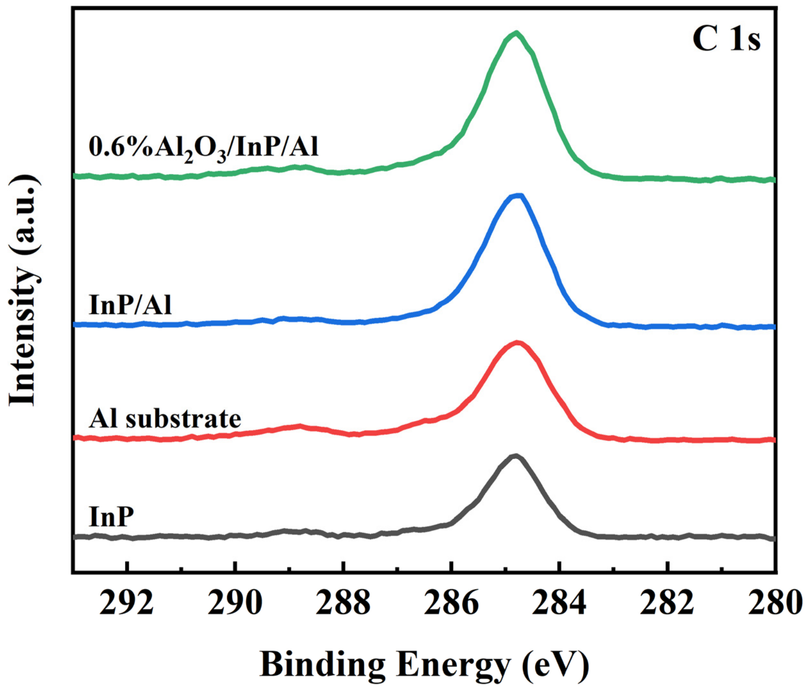

Figure S3. C 1s spectra of InP, Al substrate, InP/Al, and 0.6%Al_2O_3/InP/Al.

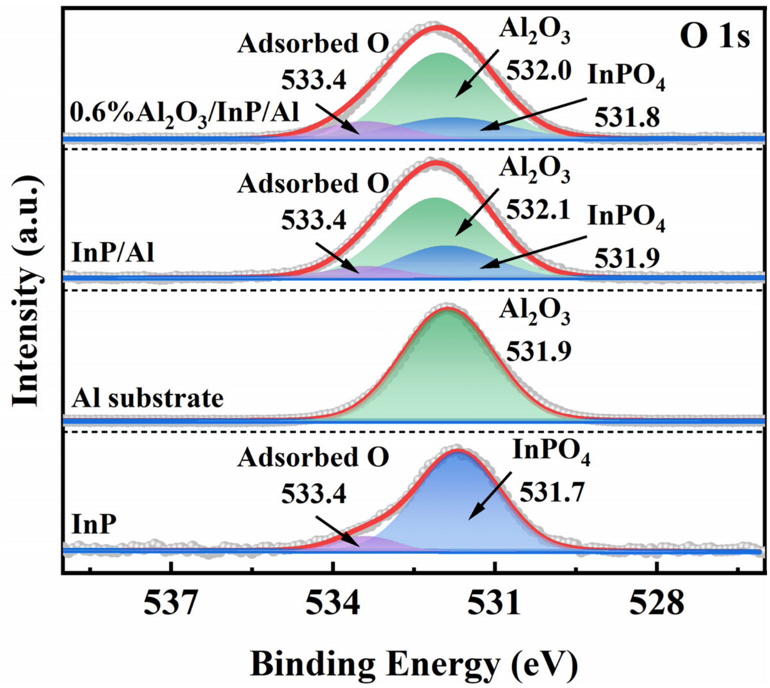

Figure S4. O 1s spectra of InP, Al substrate, InP/Al, and 0.6%Al_2O_3/InP/Al.

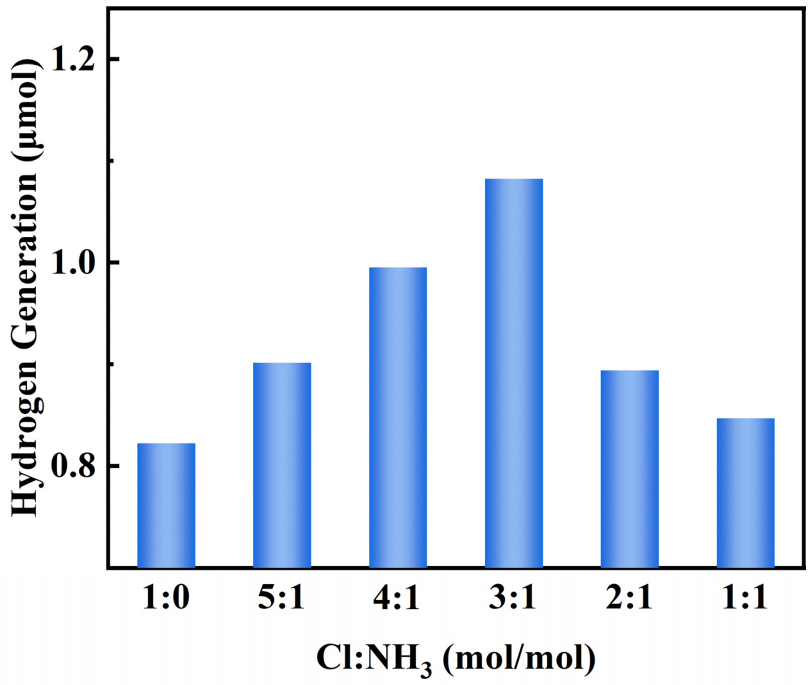

Figure S5. The photocatalytic hydrogen production of InP/Al prepared by adjusting the molar ratios between Cl (in $InCl_3$) and NH_3.

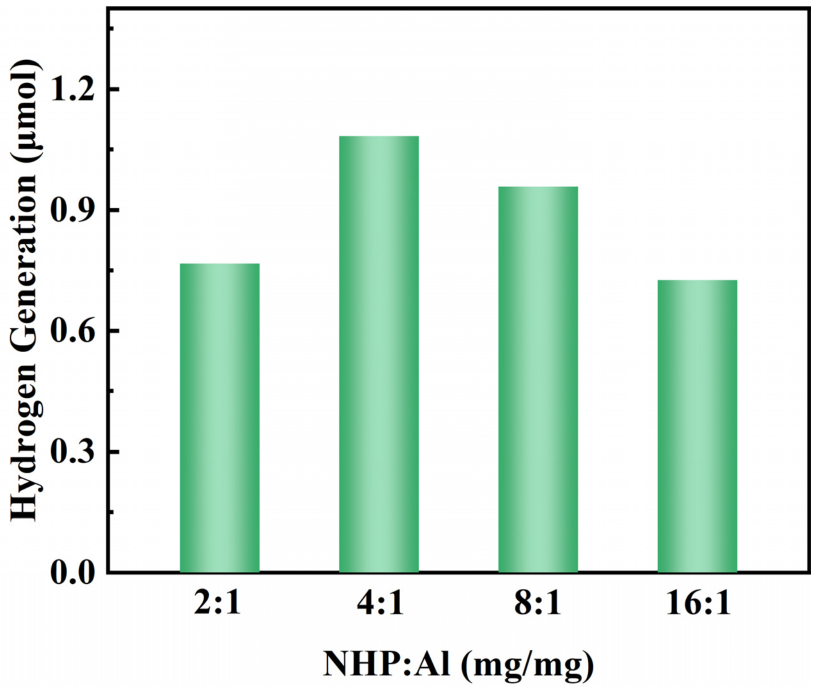

Figure S6. The photocatalytic hydrogen production of InP/Al is affected by NHP:Al mass ratio.

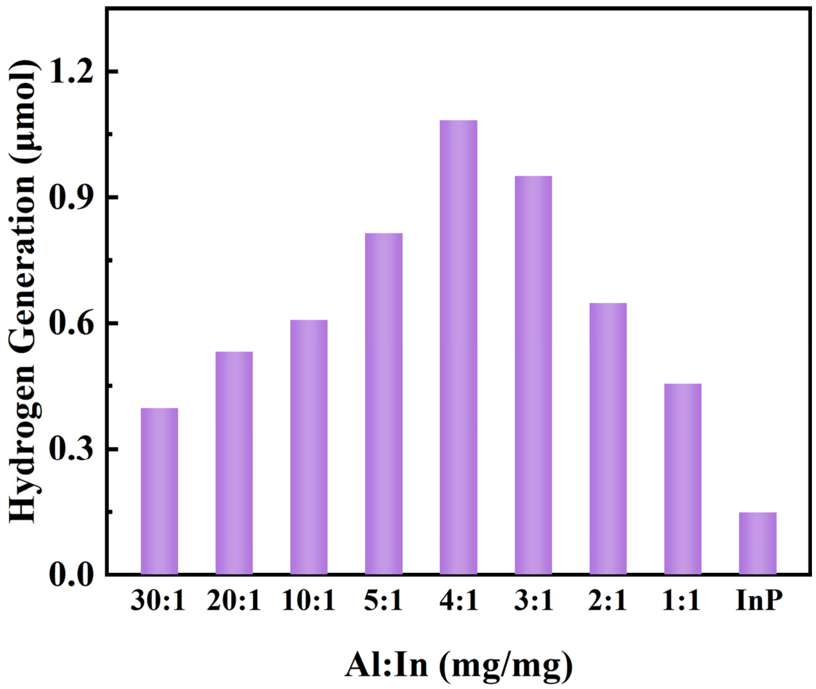

Figure S7. The photocatalytic hydrogen production of InP/Al is affected by Al:In mass ratio.

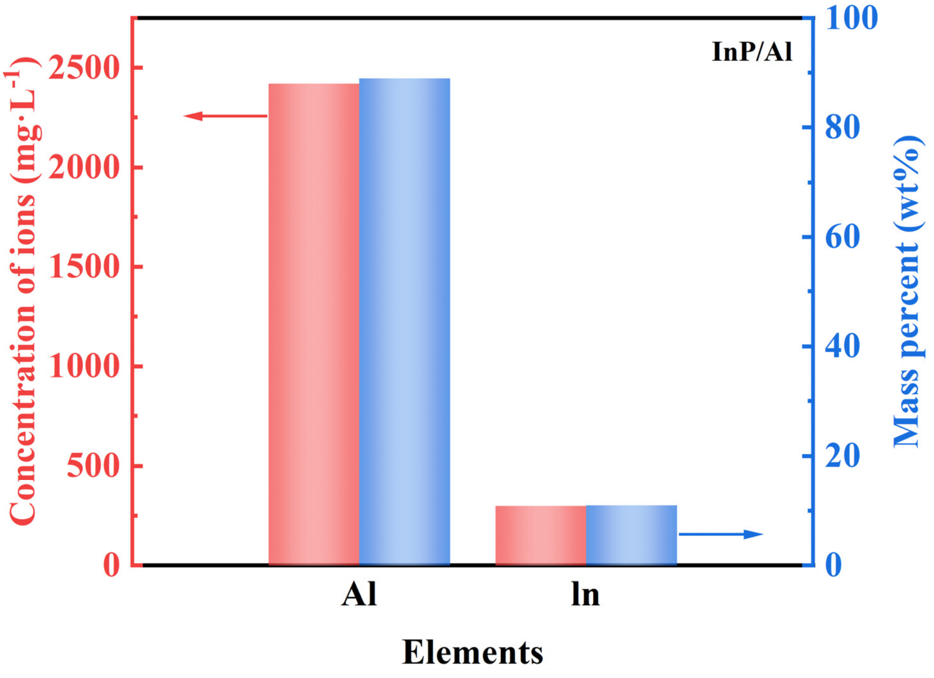

Figure S8. The content of Al and In in InP/Al.

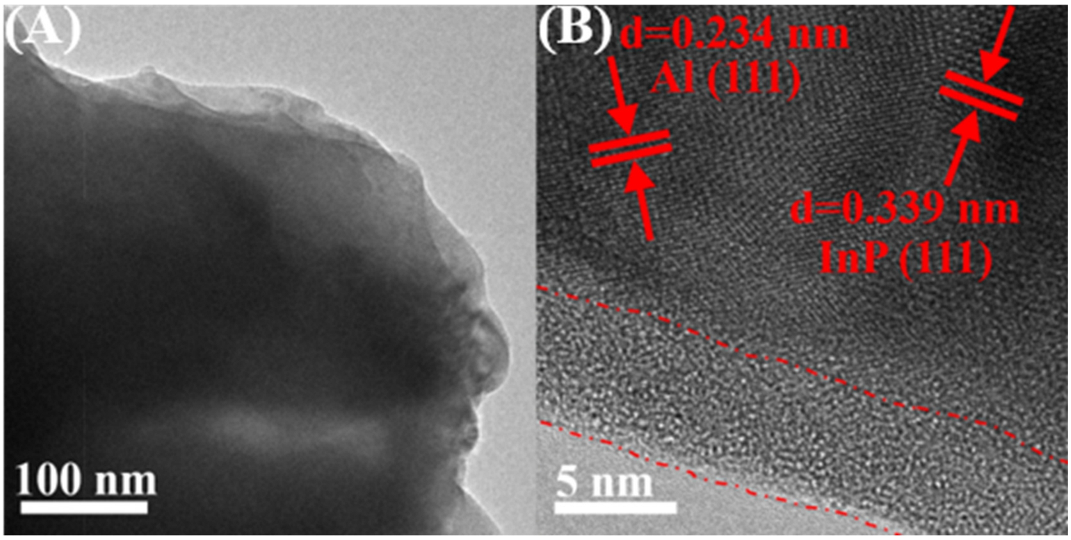

Figure S9. (A) TEM and (B) HRTEM images of 1.0%Al_2O_3/InP/Al photocatalyst.

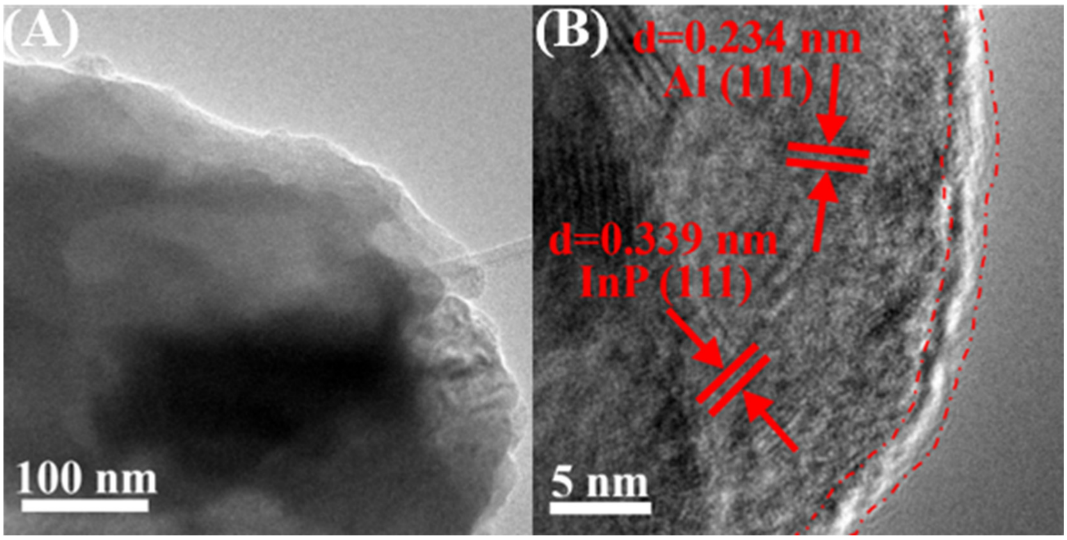

Figure S10. (A) TEM and (B) HRTEM images of 0.6%Al_2O_3/InP/Al photocatalyst after 10 hours of photocatalytic reaction.

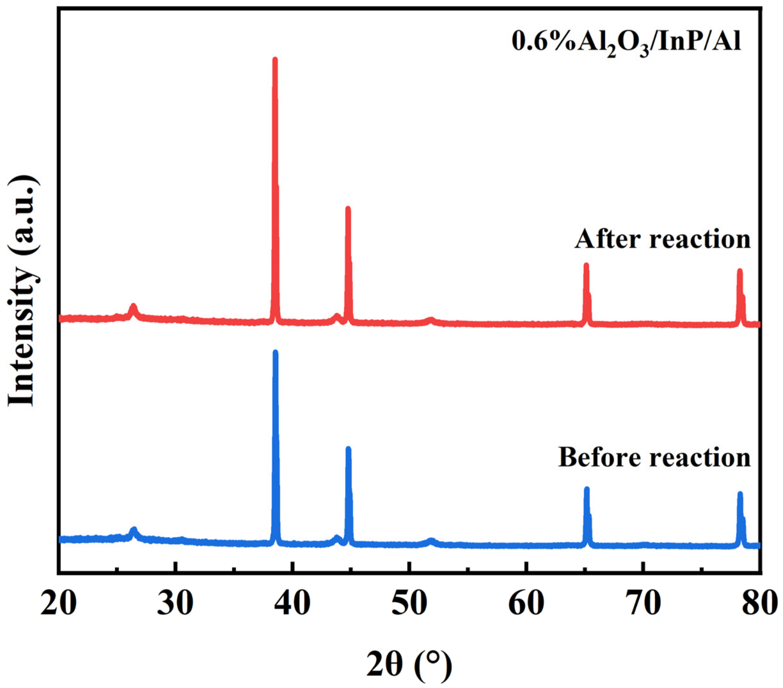

Figure S11. XRD patterns of 0.6%Al_2O_3/InP/Al photocatalyst after 10 hours of photocatalytic reaction.

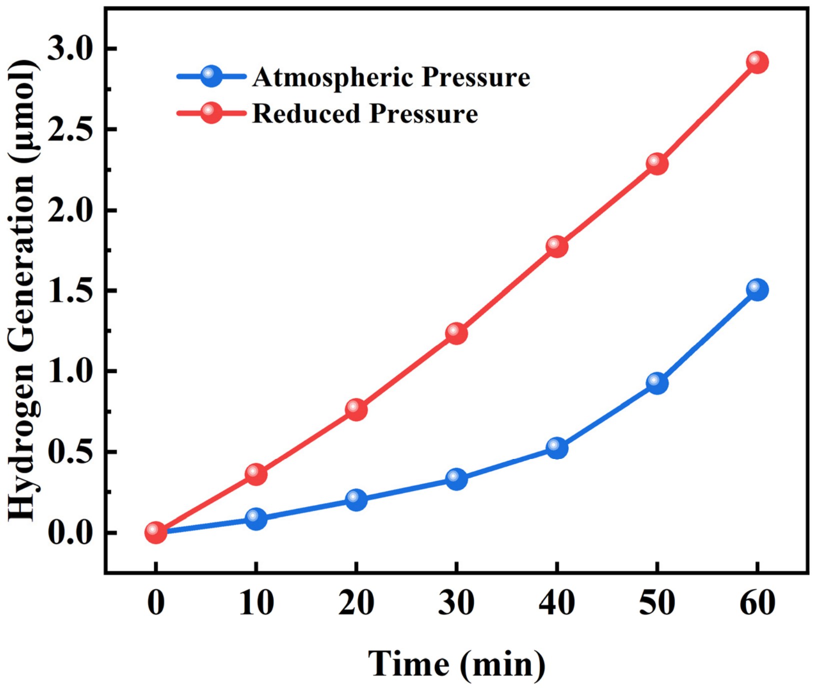

Figure S12. The photocatalytic hydrogen production activity under reduced pressure over 0.6%Al_2O_3/InP/Al photocatalyst.

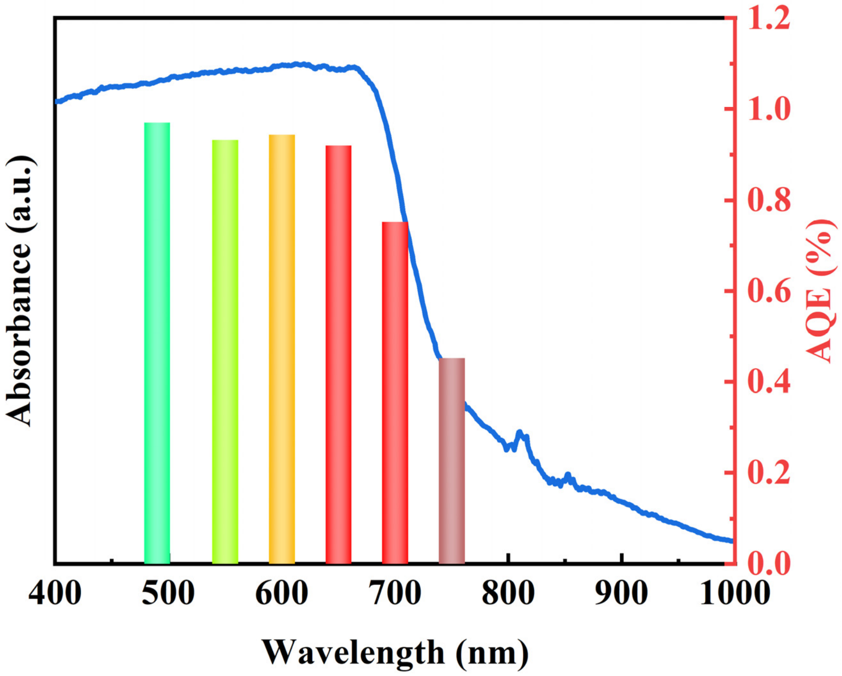

Figure S13. The AQE of 0.6%Al_2O_3/InP/Al catalyst at different wavelengths.

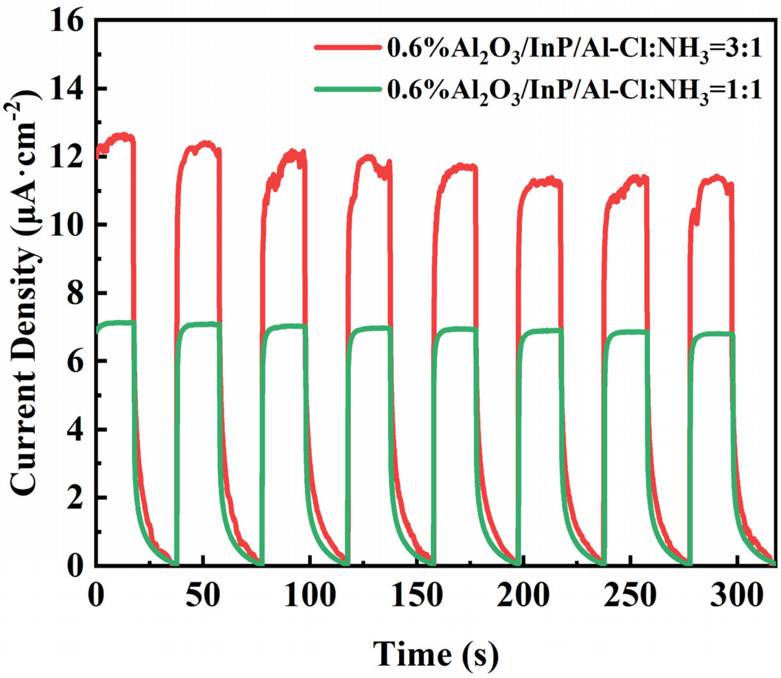

Figure S14. The transient photocurrent response of 0.6%Al_2O_3/InP/Al with varying Cl (in $InCl_3$) to NH_3 molar ratios (1:1 and 3:1).

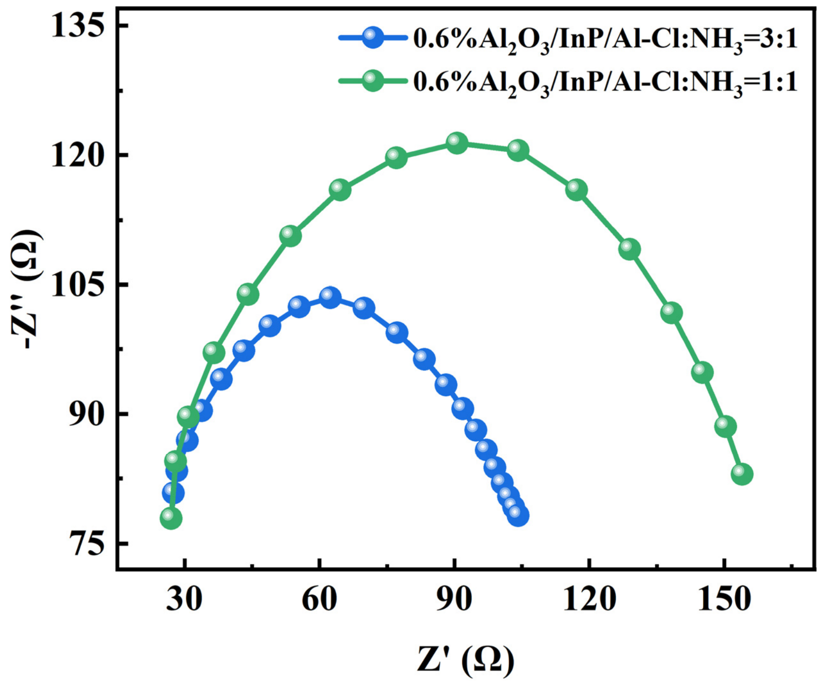

Figure S15. The EIS spectra of 0.6%Al_2O_3/InP/Al with varying Cl (in $InCl_3$) to NH_3 molar ratios (1:1 and 3:1).

Table S1 The AQEs of hydrogen production using In-based and Al-based photocatalysts.

NO.	Photocatalyst	Solution	AQE	Stability	Ref.
1	Al_2O_3/InP/Al	H_2O	0.97% at 500 nm	10 h	This work
2	$Zn_mIn_2S_{3+m}$	H_2O	0.45% at 500 nm	15 h	[1]
3	BP-$InVO_4$	H_2O	0.53% at 500 nm	15 h	[2]
4	$BiFeO_3$/$ZnIn_2S_4$	H_2O	0.06% at 510 nm	138 h	[3]
5	$Nb_4C_3T_x$ MXene@$ZnIn_2S_4$	H_2O	0.09% at 600 nm	20 h	[4]
6	Zn_i doping $ZnIn_2S_4$	H_2O	0.19% at 600 nm	32 h	[5]
7	Pt/CdS@Al_2O_3	H_2O	0.02% at 520 nm	30 h	[6]
8	Zn_3As_2/Al_2O_3	H_2O	0.35 % at 750 nm	9 h	[7]

Reference:

1. Sun, X.; Wang, Y.; Song, M.; Liu, F.; Lan, D.-H.; Yin, S.-F.; Chen, P., Local polarization redistribution in $Zn_mIn_2S_{3+m}$ for the enhancing synergetic piezo-photocatalytic overall water splitting. *J. Colloid Interface Sci.* **2024**, 665, 999-1006.
2. Yue, S.; Hu, W.; Wang, J.; Sun, M.; Huang, Z.; Xie, M.; Yu, Y., Dramatically promoted photocatalytic water splitting over $InVO_4$ via extending hole diffusion length by surface polarization. *Chem. Eng. J.* **2022**, 435, 135005.
3. Zhang, J.; Zhang, Y.; Li, L.; Yan, W.; Wang, H.; Mao, W.; Cui, Y.; Li, Y.; Zhu, X., Synergizing the internal electric field and ferroelectric polarization of the $BiFeO_3/ZnIn_2S_4$ Z-scheme heterojunction for photocatalytic overall water splitting. *J. Mater. Chem. A* **2023**, 11, (1), 434-446.
4. Liu, X.; Zhang, J.; Xu, J.; Li, Y.; Du, Y.; Jiang, Y.; Lin, K., Hydroxyl-modified $Nb_4C_3T_x$ MXene@$ZnIn_2S_4$ sandwich structure for photocatalytic overall water splitting. *J. Colloid Interface Sci.* **2023**, 633, 992-1001.
5. Sun, B.; Bu, J.; Chen, X.; Fan, D.; Li, S.; Li, Z.; Zhou, W.; Du, Y., In-situ interstitial zinc doping-mediated efficient charge separation for $ZnIn_2S_4$ nanosheets visible-light photocatalysts towards optimized overall water splitting. *Chem. Eng. J.* **2022**, 435, 135074.
6. Ning, X.; Zhen, W.; Wu, Y.; Lu, G., Inhibition of CdS photocorrosion by Al_2O_3 shell for highly stable photocatalytic overall water splitting under visible light irradiation. *Appl. Catal., B* **2018**, 226, 373-383.
7. Jia, M.; Ning, X.; Lu, G., Stable and wide spectrum response Zn_3As_2/Al_2O_3 photocatalyst for photocatalytic overall water splitting. *Int. J. Hydrogen Energy* **2024**, 51, 1366-1374.
